# Catheter ablation versus no ablation for atrial fibrillation in cardiac amyloidosis: a propensity-matched cohort study

**DOI:** 10.21542/gcsp.2026.26

**Published:** 2026-06-30

**Authors:** Joud Fahed, Mohammad Hamza, Azka Naeem, Asad Ur Rab, Muhammad Hashim Khan, Mohammad Ali Sheffeh, Jawad Basit, Salem Assiri, M Chadi Alraies

**Affiliations:** 1 Department of Medicine, Ascension Saint Agnes Hospital, Baltimore, Maryland, USA; 2 Department of Hospital Medicine, Guthrie Cortland Regional Medical Center, Cortland, NY, USA; 3 Advanced Cardiac Imaging Department, St. Francis Hospital and Heart Center, Roslyn, New York, USA; 4 Department of Medicine, Foundation University Medical College, Islamabad 44000, Pakistan; 5 Department of Medicine, Maimonides Medical Center, Brooklyn, New York, USA; 6 Department of Medicine, Henry Ford, Warren, Michigan, USA; 7 Department of Medicine, Rawalpindi Medical University, Rawalpindi, Pakistan; 8 King Fahad Armed Forces Hospital, Ministry of Defense Health Services, Jeddah, Saudi Arabia; 9 Department of Interventional Cardiology, Detroit Medical Center/Wayne State University, MI, USA

## Abstract

Background: Atrial fibrillation (AF) is extremely common in cardiac amyloidosis (CA) due to amyloid infiltration and atrial electrical remodeling. We aim to compare ablation outcomes versus no-ablation in patients taking anti-arrhythmic drugs (AAD) in CA-associated AF.

Methods: Using TriNetX database (2019-2025), 5,562 patients with CA and AF were identified and divided into two groups: catheter ablation plus AADs (*n* = 893) versus medical therapy alone (*n* = 4,669). Baseline characteristics were adjusted and 1:1 propensity score matching was performed to account for baseline differences. Primary composite outcome included all-cause mortality, ischemic strokes, bleeding and subsequent myocardial infarction.

Results: 870 patients were included in each cohort. At 12-month follow-up, catheter ablation was associated with a significantly lower risk of the composite outcome (8.3% vs 13.2%; HR 0.591, 95% CI [0.418–0.834], *p* = 0.002), and reduced all-cause mortality. There were no significant differences in all-cause hospitalization, emergency department visits, new-onset heart failure, atrioventricular block, or cardiac arrest. Repeat ablation or cardioversion, and subsequent pacemaker or ICD implantation were more frequent in the ablation group.

Conclusion: Catheter ablation was associated with improved short-term outcomes despite higher arrhythmia recurrence. Ablation may be considered in carefully selected patients, particularly those with earlier-stage disease or significant symptoms.

## Introduction

Cardiac amyloidosis (CA) is a restrictive cardiomyopathy characterized by abnormal misfolded protein disposition in the myocardium, leading to diastolic dysfunction, conduction abnormalities, and arrhythmias^1^. The two main types of CA are: light chain (AL), associated with poor prognosis, and transthyretin (ATTR) amyloidosis, which is increasingly common among the elderly population^2^.

Atrial fibrillation (AF) is the most common sustained arrhythmia worldwide and is considered a major risk factor for embolic stroke, transient ischemic attacks, and heart failure. Cardiac amyloidosis patients are at extraordinary risk for atrial fibrillation, with a prevalence up to 50% and as high as 70–80% in ATTR cardiomyopathies^3^. AF is the predominant arrhythmia in patients with cardiac amyloidosis, with the highest burden in the ATTR subtype and less commonly in the AL subtype^4^. Atrial flutter is also common in amyloidosis; meanwhile, atrioventricular blocks and ventricular arrhythmias are less common and mainly present in advanced diseases with a higher frequency than the broader population^5^.

Atrial fibrillation and flutter can also be found in isolated atrial amyloidosis in the absence of systemic involvement secondary to overproduction of atrial natriuretic peptide (ANP), especially its dimeric form^6^. The development of atrial fibrillation in these patients stems primarily from increased atrial pressures, structural expansion of the atria, heightened wall stress, and autonomic dysfunction^7^. Patients with cardiac amyloidosis face elevated risks of endocardial thrombus formation and systemic embolization^8^. Several factors contribute to this prothrombotic condition, including myocardial damage, diminished chamber blood flow, and an inherent hypercoagulable tendency. Patients are usually started on anticoagulation regardless of their CHA_2_DS_2_-VASc scores^9^.

Apart from antiarrhythmic drugs (AAD), rhythm control is usually attempted, especially in early stages and in symptomatic patients^10^. Data on AF ablation in CA are limited but growing; it is usually not recommended since the infiltrative nature of the disease decreases the chance of successful results. Some studies revealed that patients were free of atrial arrhythmias post-catheter ablation, especially if ablation is done in highly selected patients with early-stage disease^11^; meanwhile, others recommend against ablation in advanced amyloid stages^12^. Nevertheless, some recommend AV node ablation with pacemaker implantation over pulmonary vein isolation in refractory symptomatic cases^10^. Hence, we conducted a retrospective cohort study using real-world large cohort samples through TriNetX US Collaborative Network, aiming to evaluate the outcomes of atrial fibrillation catheter ablation in patients with cardiac amyloidosis and assess the outcomes of catheter ablation, including the most feared complications in practice.

## Methods

### Data sources

We conducted a retrospective cohort study using the TriNetX Global Research Network^13^, a federated research platform providing access to de-identified electronic health records from participating healthcare organizations in multiple countries. The TriNetX platform is compliant with the Health Insurance Portability & Accountability Act (HIPAA) and General Data Protection Regulation, and allows real-time analyses. The Western Institutional Review Board has granted TriNetX a waiver of informed consent since this platform only aggregates counts and statistical summaries of de-identified information. The study period extended from 2019, to 2025, and data extraction was performed on January 4th, 2026.

### Study population

110 healthcare organizations (HCOs) from multiple countries, including the United States, United Kingdom, Canada, Australia, and several countries within the European Union, were queried. Among these countries, the United States contributed the largest proportion of patient-level data, accounting for the majority of included cases. A total of 64 providers returned patient records meeting the predefined criteria.

Patients aged 18 years or older with cardiac amyloidosis who underwent treatment for atrial fibrillation were identified using both International Classification of Diseases, Version 10 (ICD-10) codes and confirmed with Current Procedural Terminology (CPT) codes. Systematized Nomenclature of Medicine) SNOMED codes were also used to define the Ablation procedure and drugs used. Table S1 shows the list of codes used to identify the patients.

**Figure 1.**
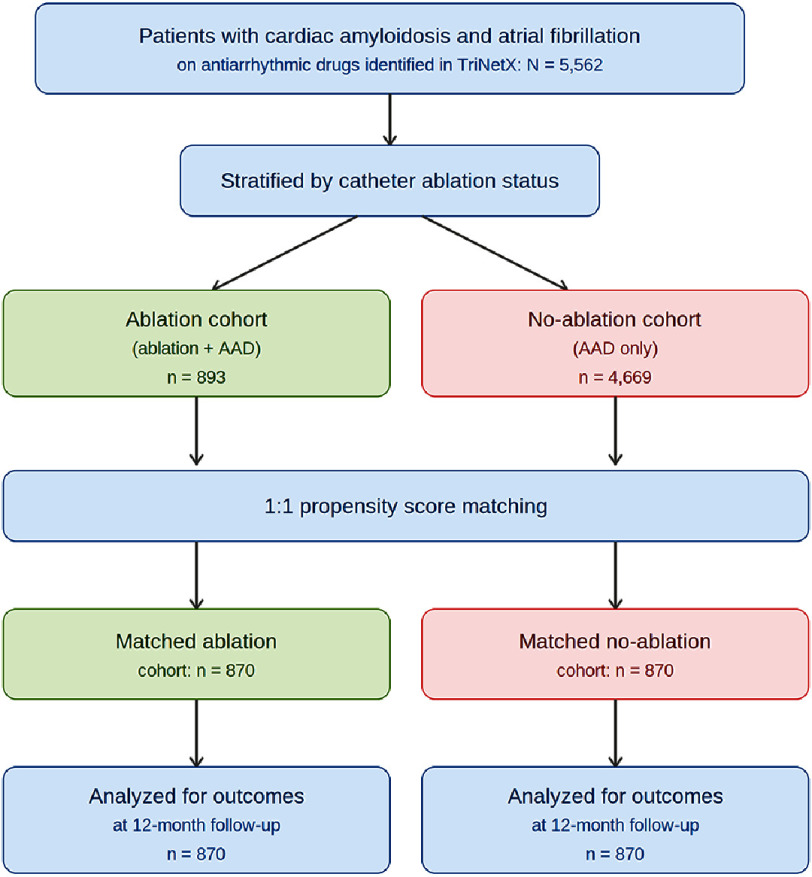
CONSORT style flow diagram of patient cohort identification, propensity score matching.

Patients with cardiac amyloidosis were defined as having cardiomyopathy identified with ICD 10 and amyloid. Included patients in the study were already on antiarrhythmic medications, including Class Ic (Propafenone, Flecainide) and Class III (Amiodarone, Dronedarone, Sotalol, and Dofetilide). Class Ic antiarrhythmics are generally not preferred in cardiac amyloidosis and usually avoided. However, eligibility was based on any guideline antiarrhythmic agent reflecting real-world prescription. Nonetheless, few patients in each cohort used them, and their use was balanced among matched cohorts. The cohort was then stratified based on undergoing AF ablation into two cohorts. An ablation cohort: patients who underwent catheter ablation for atrial fibrillation, and a no-ablation cohort: patients with atrial fibrillation and cardiac amyloidosis who did not undergo ablation. The initial sample included 893 ablation and 4,669 non-ablation patients. After 1:1 propensity score matching, 870 patients remained in each group (Figure 1).

### Outcomes

Each outcome was evaluated at a median follow-up of 1 year. The primary outcome was a composite of all-cause mortality, ischemic strokes, bleeding, and subsequent myocardial infarction (MI). Secondary outcomes included all-cause mortality, repeat ablation or cardioversion, all-cause hospitalization or emergency room (ER) visits, new-onset heart failure, complete AV block, cardiac arrest, pacemaker/ICD placement, Pericardial effusion, and tricuspid/Mitral regurgitation.

### Statistical analysis

Data for potential covariates affecting the outcomes, such as baseline demographics (age, sex, race/ethnicity), socioeconomic determinants (e.g., housing and economic circumstances), lifestyle factors, medications, and clinical comorbidities relevant to cardiac amyloidosis and atrial fibrillation (e.g., hypertension, heart failure, ischemic heart disease, and renal disease) were extracted.

**Figure 2.**
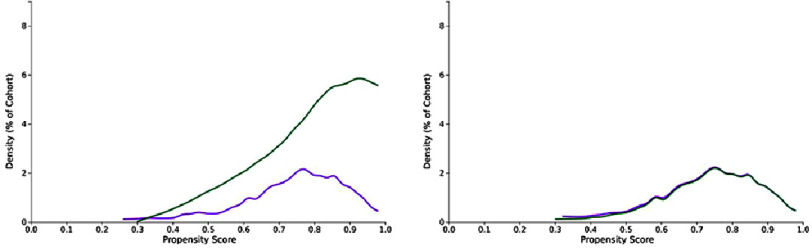
Propensity score density function - Before (left) and after matching (right). Cohort 1 - purple, cohort 2 –green.

To address potential confounding caused by the differences in baseline data, we performed 1:1 Propensity score matching using the built-in analytical functions of the TriNetX platform Figure 2. Propensity scores were generated using a logistic regression model that estimated the probability of undergoing catheter ablation based on a comprehensive set of pre-specified covariates. Matching was executed using a greedy nearest-neighbor matching algorithm with a caliper width of 0.1 pooled standard deviations of the logit of the propensity score. This algorithm pairs patients in the ablation cohort with those in the non-ablation cohort who have the closest propensity scores, ensuring that matches are made within a strict tolerance to maximize similarity. We assessed the quality of the match by calculating standardized mean differences (SMD) for all baseline characteristics. An SMD value of <0.1 was defined as the threshold for adequate covariate balance between the two cohorts. Balance diagnostics were performed before and after matching. Visual inspection of propensity score density plots was also performed to verify the overlap of distributions between groups.

Cumulative incidence was calculated using Kaplan–Meier methods with log-rank tests for between-group comparisons. Cox proportional hazards models generated hazard ratios with 95% confidence intervals. The proportional hazards assumption was verified using Schoenfeld residuals. Patients with pre-existing outcomes were excluded from respective analyses. Multivariable Cox regression modeled the primary outcome, and all-cause mortality, incorporating relevant covariates (including age, race, gender, comorbidities, medications, baseline LVEF, NT-Pro BNP, eGFR, HbA1c, etc.) regardless of univariate significance^14^.

Taking into consideration the residual age difference SMD of 0.125 post matching, which is slightly greater than our threshold of 0.1, a sensitivity analysis was performed aiming to quantify the strong unmeasured confounding that would need to explain away the primary association.

## Results

From a total of 5,562 patients with cardiac amyloidosis and atrial fibrillation identified between January 2019 and March 2025, 893 underwent catheter ablation in addition to receiving AAD, while 4,669 received AAD without ablation. After 1:1 propensity score matching, 870 matched pairs were analyzed (Figures 1–2, Table 1).

**Table 1:** Baseline characteristics before and after propensity score matching.

**Variable**	**Before Propensity Match**				**After Propensity Match**			
	**Ablation (*N* = 893)**	**No ablation (*N* = 4,669)**	**Std Diff.**	***P*-value**	**Ablation (*N* = 870)**	**No ablation (*N* = 870)**	**Std Diff.**	***P*-value**
**Demographics**								
Age at Index (years, mean ± SD)	73.6 ± 9.1	74.8 ± 10.2	0.127	0.001	73.6 ± 9.2	74.8 ± 9.8	0.125	0.009
Male (%)	74.8	70.7	0.092	0.013	74.6	74.9	0.008	0.868
Female (%)	25.2	29.3	0.091	0.014	25.4	25.1	0.008	0.868
White (%)	76.6	67.1	0.211	<0.001	76.3	77.9	0.038	0.424
Black or African American (%)	18.3	25.0	0.164	<0.001	18.5	18.0	0.012	0.804
Asian (%)	1.1	2.4	0.100	0.014	1.1	1.1	<0.001	1.000
Not Hispanic or Latino (%)	87.7	86.2	0.044	0.238	87.6	86.9	0.021	0.666
Hispanic or Latino (%)	1.5	1.9	0.037	0.338	1.5	2.3	0.059	0.219
**Comorbidities**								
Hypertensive diseases (%)	90.8	82.9	0.236	<0.001	90.6	92.0	0.049	0.308
Ischemic heart diseases (%)	73.6	65.5	0.177	<0.001	73.0	73.6	0.013	0.786
Heart failure (%)	86.6	87.2	0.020	0.670	86.6	87.2	0.020	0.670
Diabetes mellitus (%)	38.0	39.9	0.038	0.432	38.0	39.9	0.038	0.432
Chronic kidney disease (%)	51.6	51.7	0.002	0.965	51.1	51.1	<0.001	1.000
Overweight and obesity (%)	44.7	30.7	0.291	<0.001	43.6	44.8	0.025	0.595
**Medications**								
Beta Blockers (%)	93.6	92.5	0.041	0.396	93.6	92.5	0.041	0.396
Anticoagulants (%)	98.7	98.5	0.020	0.681	98.7	98.5	0.020	0.681
Loop diuretics (%)	87.8	89.3	0.047	0.327	87.8	89.3	0.047	0.327
Antilipemic agents (%)	78.7	80.2	0.037	0.440	78.7	80.2	0.037	0.440
Tafamidis (%)	15.6	16.2	0.016	0.743	15.6	16.2	0.016	0.743

Baseline characteristics were well balanced across demographics, comorbidities, medications, and laboratory parameters, with no significant differences (*p* > 0.05 for all variables). Before matching, 46 of 69 baseline characteristics were significantly different between groups; all other characteristics were significantly balanced among matched cohorts, except age, which retained a small residual SMD of 0.125, prompting the performance of a sensitivity analysis.

**Figure 3.**
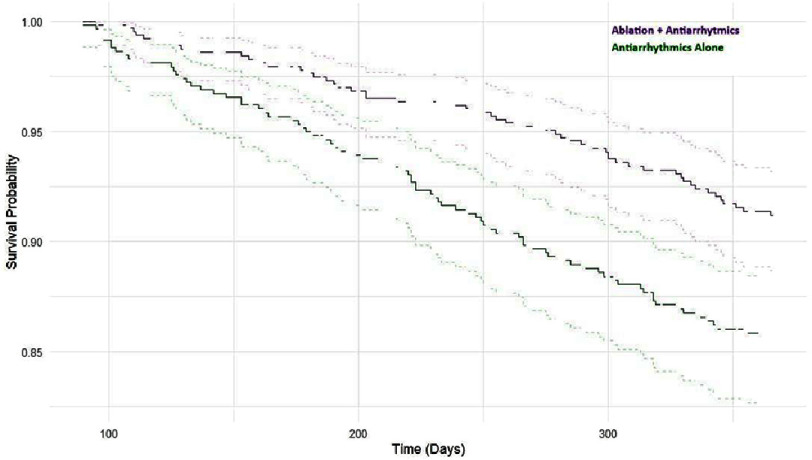
Kaplan–Meier survival analysis demonstrated a statistically significant difference in event free- survival in the composite outcome.

Primary Endpoint: At 12-month follow-up, the composite outcome occurred in 8.3% of patients in the ablation group and 13.2% of those in the non-ablation group. Kaplan–Meier survival analysis demonstrated a statistically significant difference in event-free survival for the composite outcome (HR 0.591, 95% CI [0.418–0.834],*p* = 0.002) Figure 3.

Secondary Clinical Endpoints: There was a significant difference in all-cause mortality (7.5% vs.11.9%, HR 0.586, 95% CI [0.423–0.812], *p* = 0.001). However, there were no significant differences in all-cause hospitalization or emergency room visits (46.9% vs. 40.5%, HR 1.103, 95% CI [0.956–1.272], *p* = 0.178), complete AV block (3.8% vs 2.0%, HR 1.65, 95% CI [0.885–3.075], *p* = 0.111) new-onset heart failure (26.4% vs. 27.7%, HR 0.865, 95% CI [0.722–1.036], *p* = 0.114),pericardial effusion/tamponade (4.7% vs 3.6%,HR 1.221, 95% CI [0.765–1.946],*P* = 0.402), tricuspid/Mitral regurg(17.8% vs 14.3%,HR 1.152, 95% CI [0.910–1.459],*P* = 0.240) or cardiac arrest (16.6% vs 12.3%, HR 1.243, 95% CI [0.968–1.597], *p* = 0.087).

Repeat ablation or cardioversion was significantly more frequent in the ablation cohort (9.9% vs. 4.0%, HR 2.297, 95% CI [1.551–3.403], *p* < 0.001), as was the placement of a pacemaker or ICD (7.0% vs. 3.0%, HR 2.154, 95% CI [1.261–3.679], *p* = 0.004). Clinical outcomes for both cohorts are summarised in Table 2.

**Table 2:** Clinical outcomes in ablation versus no ablation groups using hazard ratios with a confidence interval CI of 95%, and a significant *P* value < 0.05.

**Outcome**	**Ablation (*N* = 870)**	**No ablation (*N* = 870)**	**HR (95% CI)**	***P*-value**
Composite outcome	8.3%	13.2%	0.591 (0.418–0.834)	0.002
All-cause mortality	7.5%	11.9%	0.586 (0.423–0.812)	0.001
All-cause hospitalization/ER	46.9%	40.5%	1.103 (0.956–1.272)	0.178
New-onset heart failure	26.4%	27.7%	0.865 (0.722–1.036)	0.114
Cardiac Arrest	16.6%	12.3%	1.243 (0.968–1.597)	0.087
Complete AV block	3.8%	2.0%	1.650 (0.885–3.075)	0.111
Pericardial effusion/tamponade	4.7%	3.6%	1.221 (0.765–1.946)	0.402
Tricuspid/mitral regurgitation	17.8%	14.3%	1.152 (0.910–1.459)	0.240
Repeat ablation/CV	9.9%	4.0%	2.297 (1.551–3.403)	<0.001
Pacemaker/ICD placement	7.0%	3.0%	2.154 (1.261–3.679)	0.004

Sensitivity analysis: In the multivariate Cox model adjusting for age, the *E* value was 2.77 with 1.69 for the upper confidence limit and 2.80 with 1.77 for the upper confidence limit for both the composite outcome and all-cause mortality, respectively. Hence, the association was unchanged. The small, unmeasured residual age difference would need to be strong to nullify the finding. Figure S1, Table S2.

## Discussion

Data discussing catheter ablation outcomes for cardiac amyloidosis patients with atrial fibrillation remains limited in the literature given the progressive infiltrative nature of this disease and the procedure complexity with only small population studies published and controversial results. Compared to the study by Tan MC et al.^15^, our study distinguishes itself by using a significantly larger cohort sample size of 870 in each group compared to the prior relative small sample size leading to greater statistical power, more reliable, precise, robust and generalizable results. It used the most recent time frame up to 2025, capturing the up to date innovations, and advancements in catheter techniques and enhancing relevance and applicability of outcomes. Moreover, in addition to the general outcomes including mortality, hospitalizations rate and cardiac arrest; our study delved into specific catheter related outcomes including the most feared catheter related adverse events including tamponade, valve regurgitation and incidence of post procedure pacers that might prompt sometimes to favor medical therapy over invasive procedures, aiming for accurate, real-world evaluation of efficacy and safety of catheter ablation in this under-studied population, thereby enhancing clinical decision making in these scenarios.

Cardiac amyloidosis occurs when misfolded protein fibers accumulate in the heart muscle and become insoluble. The disease has two primary forms: transthyretin amyloidosis (ATTR) and immunoglobulin light-chain (AL) amyloidosis. The most prevalent type is wild-type transthyretin amyloidosis (ATTRwt), which develops from age-related accumulation of normal TTR protein. When mutations occur in the TTR gene, hereditary ATTR (ATTRv) results. AL amyloidosis develops when abnormal plasma cells generate light chains that settle in cardiac tissue^16^. The pathophysiology of atrial arrhythmias in cardiac amyloidosis is bidirectional; the amyloid fibrils’ disposition leads to increased stiffness, impaired contractility, and atrial dysfunction resulting in electromechanical remodelling. This remodelling exaggerates arrhythmogenesis through atrial dilation and conduction abnormalities^17^. Moreover, the conduction blocks caused by the amyloid deposition facilitate the re-entry circuits, premature atrial beats, and atrial fibrillation^18^. On the other hand, pathologically increased atrial wall stress caused by AF, flutter, or valvulopathy stimulates the production and secretion of ANP and subsequent amyloid disposition, perpetuating the cycle^19^.

A retrospective analysis of ATTR amyloidosis patients revealed atrial fibrillation in 70% of cases. Left ventricular ejection fraction and left atrial volume showed no significant variation, though patients with AF demonstrated higher average diastolic dysfunction grades^20^. Notably, AF patients also had reduced serum transthyretin concentrations. An observational study from Japan examined 43 cardiac amyloidosis patients—13 with AL amyloidosis and 30 with ATTR amyloidosis—and identified atrial fibrillation as the predominant arrhythmia, present in 55.8% of all participants. The occurrence rate was substantially higher among ATTR patients at 70%, compared to 23.1% in those with AL amyloidosis^21^.

Atrial arrhythmias correlate with poorer prognosis in amyloidosis patients. U.S. hospital data analysis demonstrated that cardiac amyloidosis patients with AF or AFL had elevated in-hospital mortality and greater secondary cardiac arrest risk, despite similar lengths of stay, costs, and procedural rates compared to non-amyloidosis patients^22^. These observations emphasize the importance of early detection and aggressive management of atrial fibrillation in this vulnerable group.

The restrictive cardiac physiology in CA complicates rate control, and losing atrial contraction diminishes treatment effectiveness. The American College of Cardiology advises cautious use of beta-blockers, calcium-channel blockers, and digoxin because they may suppress the compensatory heart rate increase that helps maintain cardiac output^23^. These agents have been linked to hypotension and heart failure deterioration, especially in patients who cannot augment stroke volume due to coexisting autonomic impairment. Calcium-channel blockers and digoxin carry a particularly high risk and may be poorly tolerated for biochemical reasons as well. Research by Rubinow et al. showed that digoxin binds strongly to amyloid fibrils in laboratory settings, potentially elevating tissue and serum drug levels, extending receptor exposure, and significantly raising the risk of digoxin toxicity^24^. Sotalol and dofetilide are class III antiarrhythmic agents that showed benefit in rhythm control targeting the scars and fibrosis-induced arrhythmias^25^. However, the hospitalization needs to initiate those medications, QTc prolongation, and the kidney dysfunction frequently seen in CA restricts their use. Nevertheless, class 1C agents like propafenone and flecainide are usually not recommended in structural cardiomyopathies, including cardiac amyloidosis, given their proarrhythmic effects, including atrial and ventricular re-entrant tachycardias^26^. However, evidence lacks any randomized clinical trials, especially in cardiac amyloidosis, and current recommendations are based on expert opinions^26, 27^. In our cohort, eligibility allowed any guideline antiarrhythmic agent to be included, reflecting real-world prescribing practice, with few patients only in both cohorts receiving class Ic drugs, and their use was balanced between matched cohorts and unlikely to have affected the results.

This makes catheter ablation an attractive alternative for treating atrial fibrillation in cardiac amyloidosis patients. Small patient groups have shown 1-year and 3-year freedom from recurrence rates of 75% and 60%, respectively, following AF ablation, with superior results when ablation occurs during early disease stages^28^. Among 72 ATTR-CA patients with AF, early catheter ablation decreased arrhythmia recurrence and hospitalizations while improving survival, demonstrating that timing matters significantly for ablation success^29^. However, another study documented a 80% of atrial arrhythmias recurrence rate after cardioversion, at one-year follow-up, highlighting the limited durability of cardioversion in cardiac amyloidosis^30^. Nevertheless, Petzl et al. ^31^ revealed a one-year rhythm control with catheter ablation of typical atrial flutter and left atrial arrhythmias in cardiac amyloidosis with high long-term recurrence rates. Likewise, smaller case reports indicate that pulmonary vein isolation can decrease AF burden. ACC, AHA, and HRS guidelines primarily recommend this approach for symptomatic patients^12^. However, no survival advantage has been demonstrated compared to rate control, and recurrence continues to be problematic. Our findings reflected this pattern, with the ablation group experiencing notably higher rates of repeat rhythm procedures (ablation or cardioversion), 9.9% versus 4% in the non-ablation group.

Our propensity-matched analysis of 870 patient pairs demonstrated a statistically significant reduction in the 12-month composite outcome with catheter ablation compared with medical therapy alone (8.3% versus 13.2%; HR 0.591, *p* = 0.002). A significant difference was also observed in all-cause mortality, while no significant differences emerged between groups for all-cause hospitalization, emergency department visits, or new-onset heart failure. These findings contrast with earlier research suggesting that while ablation may reduce atrial fibrillation burden, it does not consistently translate into improvements in major clinical outcomes across broader cardiac amyloidosis populations. Kaplan–Meier survival analyses demonstrated a significant improvement in event-free and overall survival, with higher 12-month survival rates observed in the ablation group compared with the non-ablation group. Altogether, along with the non-statistically significant difference in the rates of the most feared complications of catheter ablation, including pericardial effusion, tamponade, valve regurgitation, and heart block, reflect the safety of this procedure along with its improved outcomes.

The analysis demonstrated a numerical but not statistically significant increase in cardiac arrest events in patients undergoing cardiac arrest, 16.6% vs 2.3%, with HR1.243 and *p* = 0.087. Peri-procedural risk, the invasive nature of ablation compared to medical therapy, selecting patients with more advanced disease for ablation, the myocardial scar created by ablation in an infiltrative cardiomyopathy, and the subsequent risk of re-entrant arrhythmias are all possible mechanisms. However, given the non-statistically significant association and the favoritism of ablation regarding the composite outcome and all-cause mortality, these findings do not eliminate the beneficence of ablation but rather warrant rhythm monitoring and careful patient selection for ablation.

The pathophysiology of these higher repeat ablation/ ICD placement rates compared to non- cardiac amyloidosis patients can be attributed to the extensive atrial substrate modification leading to extensive fibrosis, atrial tissue disruption creating micro-reentrant circles for arrhythmias^31^. CA patients also exhibit increased non-pulmonary vein arrhythmogenic foci, a higher incidence of atrial tachycardia, and recurrent macroreentrant circuits, increasing the complexity and need for more extensive ablation procedures^32^. Nevertheless, the ongoing atrial contractility dysfunction and ischemia due to the perivascular amyloid infiltration^1, 33^ and the progressive nature of cardiac amyloidosis induce arrhythmogenesis even post successful ablation due to ongoing cardiac modulation^10, 34^. All these mechanisms increase the incidence of worse outcomes of atrial fibrillation management, including catheter ablation, compared to non-amyloid patients.

## Limitations

Our study has several limitations. First, the use of ICD-10 codes may introduce misclassification due to variability in coding practices. Second, although minimized by propensity score matching, the retrospective study design is inherently subject to potential confounding biases. Third, age retained a small imbalance SMD 0.125, *p* = 0.009 after propensity matching ; however, the primary association was not affected as per the conducted sensitivity analysis adjusting for age and *E*-value calculation. Fourth, both ATTR and AL cardiac amyloidosis subtypes were included, and subtype- or stage-specific outcomes could not be fully assessed. The median follow-up was 12 months, which may underestimate long-term recurrence of atrial arrhythmias and survival outcomes. Nevertheless, a lack of sub-analysis depending on echocardiographic characteristics of patients prior to catheter ablation, including the left atrium volume index, size, and diameter, as well as an inability to track certain numeric outcomes, including the EF, NYHA class, Pro-BNP, and creatinine post ablation, and an inability to use the database to classify based on type of ablation, like radiofrequency or pulsed field ablation.

Additionally, TriNetX collects data only from participating institutions and hospitals, introducing potential selection bias. Finally, despite multiple sites from several countries included in the TriNetX database, U.S. health care institutions contribute most patients, which can limit findings’ generalizability to non-U.S. populations or other healthcare settings.

## Conclusion

In summary, using a large cohort sample size, our study demonstrated that catheter ablation in patients with cardiac amyloidosis and atrial fibrillation was associated with improved short-term clinical outcomes, including a reduction in all-cause mortality and composite cardiovascular events, despite high rates of arrhythmia recurrence and repeat rhythm-control interventions, with no statistically significant difference in the most common feared ablation complications. While durable long-term rhythm maintenance remains challenging due to the progressive infiltrative substrate of amyloidosis, ablation appears to confer meaningful clinical benefit, likely through transient restoration of sinus rhythm and favorable hemodynamic effects. These findings support catheter ablation as a reasonable therapeutic option in carefully selected patients, particularly those with transthyretin cardiac amyloidosis at earlier disease stages or with significant symptomatic burden, when integrated into a comprehensive, individualized rhythm-control strategy.

## Funding

No funding was received for this work.

## Disclosures

The authors have no relevant relationships with industry to disclose.

## Conflicts of interests

None

## Consent to publish

The study uses de-identified patient data and thus consent to publish does not apply.
